# Functional Genomics of Epilepsy and Associated Neurodevelopmental Disorders Using Simple Animal Models: From Genes, Molecules to Brain Networks

**DOI:** 10.3389/fncel.2019.00556

**Published:** 2019-12-13

**Authors:** Richard Rosch, Dominic R. W. Burrows, Laura B. Jones, Colin H. Peters, Peter Ruben, Éric Samarut

**Affiliations:** ^1^MRC Centre for Neurodevelopmental Disorders, Institute of Psychiatry, Psychology and Neuroscience, King’s College London, London, United Kingdom; ^2^Department of Paediatric Neurology, Great Ormond Street Hospital, NHS Foundation Trust, London, United Kingdom; ^3^Department of Bioengineering, University of Pennsylvania, Philadelphia, PA, United States; ^4^Department of Biomedical Physiology and Kinesiology, Simon Fraser University, Burnaby, BC, Canada; ^5^Department of Neurosciences, Research Center of the University of Montreal Hospital Center (CRCHUM), Université de Montréal, Montreal, QC, Canada; ^6^Modelis Inc., Montreal, QC, Canada

**Keywords:** epilepsy, neurodevelopmental disorder, brain disorder, zebrafish, *Drosophila*

## Abstract

The genetic diagnosis of patients with seizure disorders has been improved significantly by the development of affordable next-generation sequencing technologies. Indeed, in the last 20 years, dozens of causative genes and thousands of associated variants have been described and, for many patients, are now considered responsible for their disease. However, the functional consequences of these mutations are often not studied *in vivo*, despite such studies being central to understanding pathogenic mechanisms and identifying novel therapeutic avenues. One main roadblock to functionally characterizing pathogenic mutations is generating and characterizing *in vivo* mammalian models carrying clinically relevant variants in specific genes identified in patients. Although the emergence of new mutagenesis techniques facilitates the production of rodent mutants, the fact that early development occurs internally hampers the investigation of gene function during neurodevelopment. In this context, functional genomics studies using simple animal models such as flies or fish are advantageous since they open a dynamic window of investigation throughout embryonic development. In this review, we will summarize how the use of simple animal models can fill the gap between genetic diagnosis and functional and phenotypic correlates of gene function *in vivo*. In particular, we will discuss how these simple animals offer the possibility to study gene function at multiple scales, from molecular function (i.e., ion channel activity), to cellular circuit and brain network dynamics. As a result, simple model systems offer alternative avenues of investigation to model aspects of the disease phenotype not currently possible in rodents, which can help to unravel the pathogenic substratum *in vivo.*

## From Phenotype to Genotype: the Era of Genetics in the Field of Neurodevelopmental Disorders

Investigating the genetic basis of childhood epilepsy and neurodevelopmental disorders over the last two decades has revealed the unexpected role of a number of key genes in guiding normal brain development and emergent brain dynamics (Myers and Mefford, 2015). Facilitated by the increasing affordability of genomic technologies, genes affecting synaptic function have been identified as causative in a diverse range of epilepsy syndromes and other neurodevelopmental disorders such that many are now considered “synaptopathies” (Grant, 2012). Interestingly, many non-synaptic genes have also been identified as risk factors in various neurodevelopmental disorders. For these genes, the underlying pathogenic mechanisms are puzzling as they are not necessarily known to regulate synaptic activity directly. Taken together, a better understanding of the functional consequences of the wide spectrum of neurodevelopmental genetic mutations is required, for which *in vivo* systems are particularly useful.

### Severe *de novo* Mutations and Genomic Alterations in Neurodevelopmental Disorders

**FIGURE 1:**
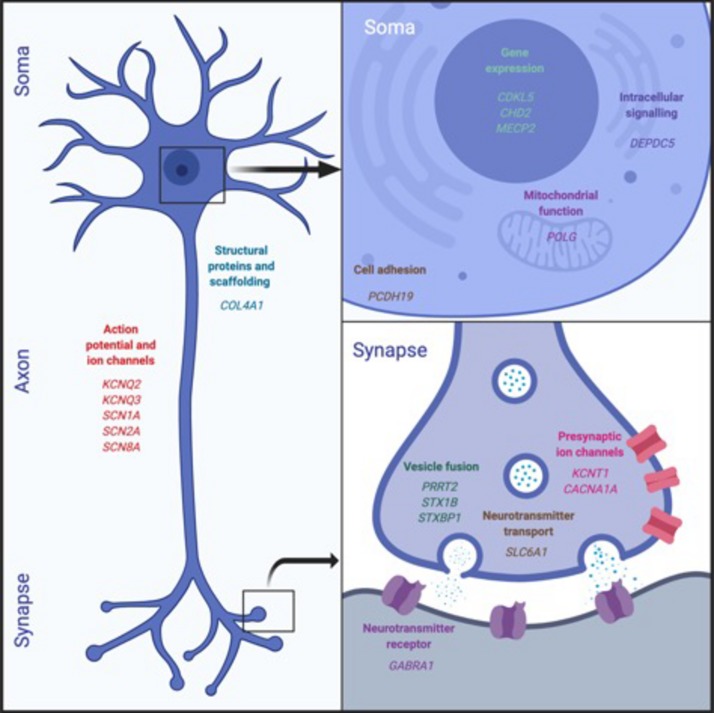
Epilepsy genes. This figure illustrates the functional classes of the most commonly identified genetic mutations in children with DDEs. These affect a broad range of neuronal functions, ranging from gene expression and intracellular signaling to neurotransmission.

Genetic insights have been particularly transformative in our understanding of some of the most severe disorders of neurodevelopment, known now as developmental and epileptic encephalopathies (DEEs) (Scheffer et al., 2016). DEEs usually occur as isolated cases in families, yet in a large proportion of cases, causative *de novo* mutations in single genes can now be identified from clinical genetic diagnostics (Epi et al., 2013; Oates et al., 2018). The most common genes and their functional effects are illustrated in Figure 1. Affordable technology that allows the identification of even small structural genomic alterations [i.e., copy number variations (CNVs)] was key to investigating the genetic basis for common neurodevelopmental disorders. Beginning with transformative studies of people living with autistic spectrum disorders (ASD) (Sebat et al., 2007; Pinto et al., 2010), we have come to understand that individually rare CNVs account for a significant proportion of the incidence not only of autism, but intellectual disability (Cooper et al., 2011), “idiopathic” generalized epilepsies (Mefford et al., 2010; Addis et al., 2016) and schizophrenia (Stefansson et al., 2008; Marshall et al., 2017), particularly where there is overlap between these conditions. Interestingly, genes identified from genome-wide association studies of particular disorders often overlap across disorder categories (Fromer et al., 2014; International League Against Epilepsy Consortium on Complex Epilepsies, 2014; Schizophrenia Working Group of the Psychiatric Genomics Consortium, 2014; Turner et al., 2017), suggesting that many of the genes have a broad neurodevelopmental role that may result in a range of recognizable syndromes or phenotypes.

### Genotype-Phenotype Correlations

The increasing ability to make genetic diagnoses at the level of individual patients carries the promise of allowing the development of targeted therapies informed by underlying pathomechanisms. However, with increasing diagnosis, the phenotypic spectrum widens and linking genotypes to phenotypes is becoming more and more challenging. Indeed, mutations in the same gene (or even identical same genetic mutations) may cause very different phenotypes in different patients. For example, up to 50% of patients with *de novo* mutations in known epilepsy genes do not have seizures (Deciphering Developmental Disorders, 2017). Another example is the spectrum of epileptic phenotypes caused by different mutations affecting the same GABA receptor gene (*GABRA*1). This spectrum ranges from some of the most severe developmental DEEs of infancy, to juvenile onset treatable generalized epilepsy syndromes (Johannesen et al., 2016). Some variability in the mapping between affected genes and phenotype can be explained by differences in specific mutations’ effects (even at the level of the same gene) on protein function (Ben-Shalom et al., 2017). This genotype-phenotype relation may be addressed in the future by increasing efforts to investigate the functional effects of individual genes as well as individual mutations *in vivo* through translational research (Scheffer et al., 2016). This gene has been described as both an epilepsy, and an autism gene but also emerges in a range of other neurodevelopmental contexts suggesting potentially shared mechanisms. Understanding the contribution of the genetic alteration to the various phenotypes is essential to now attempt and translate these broad insights into novel, targeted therapies.

## From Gene Mutation to Molecular Dysfunction (Temporal Microscale)

In the light of the difficulties in relating newly diagnosed genetic variants with their underlying functional consequences, and because of the unclear correlation between phenotype and genotype we described above, there is a need for *in vivo* models to explore the functional effects of specific genetic alterations. In particular, epilepsy has been studied using multiple model organisms, most traditionally rodents (Seyfried and Glaser, 1985; Yu et al., 2006). Although rat and mouse models have been foundational to the field, recent research has expanded to include non-mammalian models such as round worms, zebrafish, and fruit flies (Baraban, 2007; Cunliffe et al., 2015).

*Drosophila melanogaster* has become an increasingly popular model organism in epilepsy research due to its small size, short generation time, and the relative ease of stock maintenance and mutant isolation (Bier, 2005; Song and Tanouye, 2008; Cunliffe et al., 2015). These factors, in addition to the large percentage of conserved human disease genes in *Drosophila* (Fortini et al., 2000; Rubin et al., 2000; Bier, 2005), result in it being an extremely cost-effective model system for epilepsy research (Baraban, 2007; Song and Tanouye, 2008; Cunliffe et al., 2015). Developments in genome editing technology have facilitated the introduction of human disease-causing mutations into the corresponding genes of *Drosophila*, resulting in the improved ability to characterize gene-phenotype relationships as well as to perform high-throughput *in vivo* drug testing (Stilwell et al., 2006). These techniques have advanced the identification of disease-specific epilepsy treatments (Griffin et al., 2018). One example is the study of Dravet syndrome (DS), a severe form of infant-onset febrile epilepsy that is often co-morbid with other developmental disorders. DS patients are typically pharmaco-resistant (Chiron, 2011; Dravet, 2011; Griffin et al., 2018), with many common antiepileptic drugs even aggravating their seizures (Guerrini et al., 1998; Chiron, 2011; Nissenkorn et al., 2019). Thus, there exists a demand for increased therapeutic treatment options, an issue that is further complicated by the multitude of different DS-causing mutations (Meng et al., 2015; Schutte et al., 2016). Electrophysiology research has revealed that these mutations exhibit considerable variation in their channel characteristics (“channotype”), ranging from gain-of-function to loss-of-function effects (Escayg and Goldin, 2010; Meng et al., 2015; Peters et al., 2016). Elucidating the molecular variations behind this functional heterogeneity can inform drug selection for preliminary pharmacological testing, which can in turn provide *in vivo* validation for electrophysiology results. Combining these two techniques can therefore be a powerful approach to better understanding and treating DS, serving as informative steps on the pathway to clinical drug testing.

A common mutation target for generating DS models is the *Drosophila para* gene, encoding the voltage-gated sodium channels and corresponding to the human *SCN1A* gene in which many DS-causing mutations have been identified (Dravet, 2011). The *Drosophila para* gene is edited using CRISPR-cas9 to reproduce specific human *DS* causing mutations in *SCN1A*, whilst also introducing a marker mutation (e.g., eye color). In these flies, transient seizure-like behaviors [falling into their backs or sides and beginning to twitch their legs and wings, sometimes accompanied by abdominal curling (Sun et al., 2012; Schutte et al., 2016; Griffin et al., 2018)] can be induced by hyperthermia (Supplementary Video S1). Once a model organism line is validated, potential drug therapies can be assessed by mixing the therapeutic target of interest into liquified cornmeal food and allowing the flies to feed on it before subsequent seizure assays (Sun et al., 2012). Thus, modeling epilepsy with *Drosophila* enables researchers to use a simple model to shed light on the functional characterization of genetic data and to perform large-scale screenings of antiepileptic drug candidates, providing a cost-effective form of preclinical testing.

*Danio rerio* (zebrafish) is another model appropriate for linking genotype to phenotype in patient models of neurodevelopmental disorders. A major reason for this is the phylogenetic proximity of this vertebrate model, the high homology of the zebrafish and human genomes (∼80% homology), and the ease of genetic manipulation in the zebrafish. In fact, genetic modification in zebrafish is highly efficient, allowing comprehensive *in vivo* studies even of neurodevelopmental disorders with complex genetic backgrounds. One example is a recent study in which 132 schizophrenia risk variants were generated using CRISPR-Cas9 (Thyme et al., 2019). A wide array of zebrafish models have also been developed across epilepsy genes, and genes associated with broader neurodevelopmental phenotypes [e.g., *scn1lab* (Baraban et al., 2013), *gabrg2* (Liao et al., 2019), *GABRA1* (Samarut et al., 2018), *mecp2* (Pietri et al., 2013), *grin2*a/b (Thyme et al., 2019), and others]. Importantly, despite different brain anatomy and physiology to mammalian counterparts, various zebrafish models exhibit phenotypes analogous to corresponding rodent models and clinical phenotypes. In particular a zebrafish model of DS carrying a loss-of-function mutation in the *scn1lab* gene, exhibits spontaneous electrographic abnormalities reminiscent of seizures (Baraban et al., 2013). Such recordings, obtained from field electrodes placed in the midbrain of agar immobilized larval zebrafish, appear as brief, small amplitude inter-ictal like events and prolonged, multi-spike ictal-like discharges which are qualitatively comparable to epileptiform discharges in patients and mammalian epilepsy models. Furthermore, *GABRA1*^–/–^ and *gabrg2*^–^*^/^*^–^ larvae, both modeling mutations reported in common epilepsy syndromes (Wallace et al., 2001; Johannesen et al., 2016), exhibit reflexive seizure-like events in response to light stimulation, reported as convulsive motor abnormalities and abnormal brain synchrony (Supplementary Video S2; Samarut et al., 2018; Liao et al., 2019).

It is, however, important to note that zebrafish lack a cortex and therefore qualitative homologies between zebrafish and human epilepsy (a putative cortical pathology) may be more useful for broad functional characterizations of genetic epilepsies, while specific seizure subtypes may be better modeled by more complex model organisms systems. Nonetheless, given that common anti-epileptic drugs correct electrographic, and motor abnormalities in these zebrafish models, the underlying neuropathology is likely to be conserved in genetic models (Baraban et al., 2013; Samarut et al., 2018; Liao et al., 2019). Finally, zebrafish larvae are also highly amenable to high-throughput behavioral drug screens, which have already identified novel drugs for the treatment of DS, thus closing the loop from fish tank to bedside (Griffin et al., 2018).

Therefore the larval zebrafish can provide realistic models of a wide array of neurodevelopmental disorders which may open alternative avenues for investigation at scales not possible in its mammalian counterparts making them complementary models.

## From Gene Mutation to Brain Network Perturbation (Temporal Macroscale)

As discussed above, in order to translate an improved genetic understanding of neurodevelopmental disorders into novel therapies, a detailed understanding of pathomechanisms is required. Simple animal models can enable the characterization of the functional consequences of genetic mutations at multiple different scales, ranging from single-cell behavior to whole-brain dynamics, and allow this translation much more rapidly and at times more comprehensively than in mammalian models.

**FIGURE 2:**
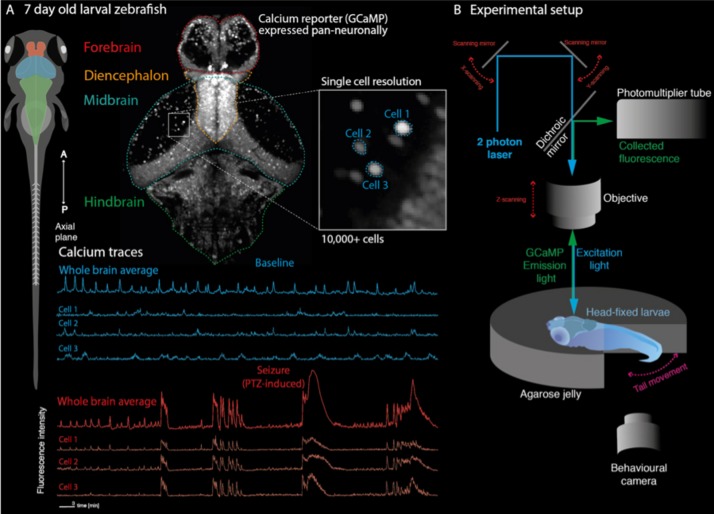
Recording whole-brain dynamics at single-cell resolution in zebrafish models of neurodevelopmental disorders. **(A)** Larval zebrafish at 7 days post fertilization are freely behaving and have all the major anatomical subdivisions of the vertebrate brain (left). Transgenic lines expressing genetically encoded calcium indicators in neurons can be used to record neuronal function through fluorescence signals. Because of their small size, the whole brain can be captured at single cell resolution (top). This allows recording of whole brain dynamics alongside single-cell behavior (bottom). **(B)** Zebrafish larvae can be embedded in transparent agarose, allowing *in vivo* imaging using fluorescence microscopy (shown here is a two-photon microscopy setup). Depending on the experimental paradigm, behavioral output can further be tracked using recordings of tail movements in tail free set ups. This allows e.g., linking of convulsive movements and brain hypersynchrony to identify epileptic seizures in the zebrafish.

The utility of simple model systems for bridging this gap is particularly evident in epilepsy. Given that seizures are an emergent property of microcircuits, understanding the effect of specific genetic mutations at a network level is necessary to explain the emergence of clinical phenotypes. At this juncture, the larval zebrafish is a particularly appealing model for studying brain network dynamics due to its amenability to whole brain imaging at single-cell resolution, allowing identification of abnormal dynamics at multiple scales (Ahrens et al., 2013). The larval fish at 7 days-post-fertilization has a small, simple brain (100,000 cells, <1 mm^3^) but is capable of a variety of complex behaviors whose brain dynamics can be monitored accurately (Figure 2). Various genetic lines of pigment deficient larval zebrafish have been developed which enable unrestricted optical access into the developing brain (Antinucci and Hindges, 2016). Furthermore, the development of various transgenic reporters of cellular activity, such as GCaMP and RGECO enable the imaging of calcium dynamics in single cells and whole brain networks during behavior, using fluorescence microscopy (Figure 2; Walker et al., 2013; Wolf et al., 2017; Chen et al., 2018). In fact, various transgenic lines have also been developed to monitor specifically GABA (Marvin et al., 2019) or glutamate (Marvin et al., 2013) signaling *in vivo*. Given that these cellular reporters have been utilized to characterize neuronal function across brain scales [from synapses to cell populations and brain networks (Walker et al., 2013; Boulanger-Weill et al., 2017; Betzel, 2018)], functional imaging of zebrafish genetic models may provide a unique window into the multi-scale functional consequences of upstream channel abnormalities. Thus, it may provide an explanatory bridge between gene mutation and whole brain clinical phenotypes.

While at present, the majority of zebrafish functional imaging studies have characterized acute, induced seizures using the GABA-A antagonist pentelynetetrazole (PTZ), a variety of useful network features have been identified which may provide insight into future genetic models. Multiple studies have reported increased functional connectivity across local and distributed brain regions during seizure events (Figure 2A; Diaz Verdugo et al., 2019; Liu and Baraban, 2019), in accordance with reports of increased phase locking in EEG recordings during seizures (Meisel et al., 2012).

Importantly, single cell-level information can be harnessed from functional imaging data to explain seizure network dynamics. For example, zebrafish imaging suggests that seizures emerge as cellular ensembles, which are composed of more spatially distant cells than pre-seizure (Diaz Verdugo et al., 2019; Liu and Baraban, 2019). Furthermore, the role of cell subtypes in the emergence of network abnormalities may be probed with the application of double transgenic larval zebrafish, expressing calcium reporters and specific cellular subtype reporters (Lyons et al., 2003; Xi et al., 2011; Shimizu et al., 2015). Such approaches have demonstrated that astrocytes facilitate widespread neuronal synchrony during generalized seizures, thereby enabling seizure state transitions (Diaz Verdugo et al., 2019). Identifying critical cell subpopulations in this way in genetic epilepsies has the potential to identify novel treatment targets in patients. Interestingly, model-based approaches which are widely used to explain network phenomena in EEG data can also be applied to calcium imaging data to test causal mechanisms underlying network features of seizures (Rosch et al., 2018). Such approaches have shown that acute seizures are caused by parameter changes in local excitation-inhibition balance, and alterations in timescales of excitatory and inhibitory connectivity. In this way the cellular mechanisms underlying observed network features in functional imaging data can be uncovered, to provide a conceptual bridge to explaining EEG phenomena during seizures, such as hypersynchrony and transitions between network states. As more genetic lines become available (Baraban et al., 2013; Samarut et al., 2018; Swaminathan et al., 2018; Liao et al., 2019), such imaging approaches may be harnessed to link gene mutation with network perturbation.

Flies have also become convenient models to perform neuronal cell recording in the adult brain (*ex vivo*) that can be studies in the context of genetic and/or pharmacological manipulations (Gu and O’dowd, 2007; Roemmich et al., 2018). As an example, electrophysiological studies carried out in adult flies that were genetically modified to mimic DS were pioneers in showing the link between pathological missense mutations and disturbances of sodium ion current activity at the receptor level (Sun et al., 2012; Schutte et al., 2014). These cellular experimentations in a genuine *in vivo* context are very advantageous in order to unravel the basic cellular mechanisms of brain circuit function and malfunction. They can also be suitable for evaluating the mechanism of action of candidate therapies against neurodevelopmental disorders that were first identified through behavioral assays.

Remarkably, the larval zebrafish is also a suitable model for longitudinal studies of neural development. Indeed, the transparency of the embryo allows one to follow *in vivo* organogenesis, in particular the observation of central nervous system structures with a single-cell resolution. Moreover, there is a large repertoire of available transgenic lines expressing fluorescent reporters in different neural cell populations such as post-mitotic neurons (huc/elavl3^+^) (Park et al., 2000), GABAergic interneurons (dlx5/6^+^) (Zerucha et al., 2000), glutamatergic neurons (vglut2a^+^) (Kimura et al., 2006) or oligodendrocytes (olig2^+^) (Shin et al., 2003). This is of a particular interest in the context of neurodevelopmental disorders for which one can expect defects in brain wiring to occur during early neurodevelopment. In this context, the accessibility of the zebrafish embryo from the earliest stages of development is an advantage compared to mammals in which the embryos develops *in utero*. The use of larval zebrafish has proven particularly useful in modeling several human neurological disorders with a developmental component such as ASD or epileptic encephalopathy. For example, using specific transgenic lines identifying excitatory versus inhibitory neuronal networks, Hoffman et al. (2016) revealed a specific deficit of GABAergic neuronal population networks in the forebrain of zebrafish larvae mutant for *CNTNAP2*, an ASD-related gene. Another zebrafish model of ASD (shank3b^–/–^) displays a reduction in the overall brain neuronal content as revealed by a transgenic line expressing a fluorescent protein in post-mitotic neurons (Liu et al., 2018). Interestingly, the development of these neuronal populations can be followed over time and the defects can therefore be monitored throughout neurodevelopment. More recently, two genetic models of epilepsy [idiopathic generalized epilepsy: *GABRA*1^–/–^ (Samarut et al., 2018), and focal epilepsy: depdc5^–/–^ (Swaminathan et al., 2018)] have been generated. These models depict relevant phenotypes to the human disorders, but more interestingly, they demonstrated impaired GABAergic synaptic network branching in the mutant larval brains identifying a potential pathomechanism. In this way, zebrafish genetic models can be harnessed to further understand the developmental component of these diseases and in so doing, at least partially, account for the pathogenicity of the mutations tested.

As a result, simple models like flies and zebrafish appear to be an amenable model to (i) mimic human genetic condition associated with neurological disorders, (ii) investigate the consequence at the neuronal network activity level through *in vivo* calcium imaging, and (iii) unravel neurodevelopmental defects associated with the disorder.

## Conclusion: Simple Models as Genetic Avatars of Human Patients for a Systemic Approach (From Gene to Physiology)

In the current context of fast-evolving accessibility to genetic diagnosis, more and more genetic basis of neurological disorders are being unraveled. They opened the door to a new challenges that is the translation of this genetic data into functional read-out. Can we predict the functional consequence of a specific mutation in a particular gene? What are the effects of a specific mutation on the activity of the protein? at the level of the neuronal network? At the scale of whole neurodevelopment? These puzzling questions necessitate the use of fast and complementary *in vivo* approaches. In this review, we are discussing how simple animal models can be employed to bridge the gap between genetic diagnosis and functional studies. Considering the fast development of mutagenesis techniques that now allow to mimic a specific genetic mutation in these simple models, plus their relative low-cost of housing as well as their fast generation time, they represent a model of choice to study neurodevelopmental disorders in an integrated fashion and at multiple scales. Thanks to their versatility, these simple animal models can unravel the basic pathomechanims of gene mutations and therefore open new avenues for therapy development. As mentioned previously, they are also convenient for standardize procedures, in particular for high-throughput screening of small molecules. Interestingly, they are also very convenient for genetic manipulation. Indeed, by microinjecting molecular tools in the one-cell stage embryo, it is possible to either knockdown [with morpholinos or CRISPRi (Long et al., 2015)], knockout [by CRISPR/Cas9 genome editing (Hwang et al., 2013)] or overexpress [by CRISPRa (Long et al., 2015) or by injecting *in vitro* transcribed messenger RNAs or transposon plasmids for transgenesis] the expression of a gene of interest. As a result, these simple animal models can also serve as tools to test genetic-derived therapeutic strategies through restorative functional assays after modulating the expression of candidate genes.

## Author Contributions

RR, DB, LJ, PR, and ÉS wrote the manuscript. CP performed the *Drosophila* recording. ÉS performed the fish recording.

## Conflict of Interest

ÉS is a co-founder of Modelis Inc.

The remaining authors declare that the research was conducted in the absence of any commercial or financial relationships that could be construed as a potential conflict of interest.

## Supplementary Material

VIDEO S1 Temperature-induced seizures in *Drosophila* with a mutation in the *Para* gene, analogous to *SCN1A*. To introduce the human A1273V mutation into the corresponding location on *Drosophila*’*s* para gene (A1356V), we used the *Drosophila* CRISPR-Cas9 system developed by a set of labs at Brown and University of Wisconsin https://flycrispr.org/about/ (citation). We designed 2 RNA ides and a donor vector with mutated template DNA for homology-directed repair as well as insertion of a DsRed eye reporter in the subsequent intron. Embryo injections and genetic crosses were performed by the company BestGene, Inc. (Chino Hills, CA, United States).

VIDEO S2 Light-induced seizures in zebrafish with a mutation in the *GABRA1* gene. As described previously in Samarut et al. (2018). Infrared camera recording of 5, 5 wpf *GABRA*1^+/+^(top, *n* = 4), ±(middle, *n* = 4), and −/− (bottom, *n* = 4) embryos maintained in a 12-well plate, using a DanioVision recording chamber. Light was switched on about 5 s after the video started (see icon on the top left corner). Immediately after light turning on, the −/− juveniles underwent a first phase of tonic-like seizures lasting few seconds and during wish they convulsed heavily and lost their posture. After a short pause, they underwent a second clonic-like phase that lasted up to 1 min and during which they swam quickly in circle (the “whirlpool” phenotype).
